# Tracheal metastasis of small cell lung cancer

**DOI:** 10.4103/0970-2113.56358

**Published:** 2009

**Authors:** Sajal De

**Affiliations:** *Department of Pulmonary Medicine, Bhopal Memorial Hospital & Research Centre, Bhopal-462038, India*

**Keywords:** Endotracheal metastasis, small cell lung cancer, tracheal tumor

## Abstract

Endotracheal metastases of primary lung cancer are rare. Only one case of tracheal metastasis from small cell lung cancer has been reported in literature. Here, we report a rare case of a 45-year-old woman who was admitted for sudden-onset breathlessness with respiratory failure and required ventilatory support. Endotracheal growth was identified during bronchoscopy, and biopsy revealed endotracheal metastasis of small cell lung cancer.

## INTRODUCTION

Small cell lung cancer (SCLC) accounts for approximately 20% to 25% of all lung cancers. This type of tumor originates predominantly in the submucosal and peribronchovascular connective tissue. SCLC exhibits aggressive behavior and has a tendency for early dissemination; it commonly involves lymph node, liver, adrenal, bone, and central nervous system (CNS). Endobronchial obstruction by SCLC is rare and is due to progressive circumferential compression.

Metastasis in trachea is unusual and occurs mostly from extrapulmonary malignancies.

## CASE REPORT

A 45-year-old woman was shifted to our hospital on invasive ventilatory and inotropic support. Her relatives informed that she had history of progressive dry cough for the last one year and was receiving bronchodilators and other supportive therapies. Her symptoms were aggravated for the last 15 days. Two days prior to this visit to our hospital, she developed sudden onset of severe breathlessness and became hypoxic. She was intubated and put on ventilatory support. Left-sided pneumonia was suspected, and fiber-optic bronchoscopy was done. On bronchoscopy, tracheal growth was detected. Subsequently, she was referred to our hospital to treat the airway obstruction.

On general physical examination, she was drowsy, her blood pressure was 90/60 mmHg, and pulse rate was 116/ min. There was no clubbing, and she had no significant lymphadenopathy. Respiratory system examination showed bilateral diffuse coarse inspiratory crackles.

She was resuscitated, and ventilatory support was continued. Laboratory examination showed hemoglobin - 8.6 g% and total leukocyte count - 12,400/μL (neutrophil - 87%, lymphocyte - 08%, eosinophil - 03%, and monocyte - 02%). Her kidney function, liver function, and serum electrolyte were normal. Her HIV status was negative.

**Figure 1:**
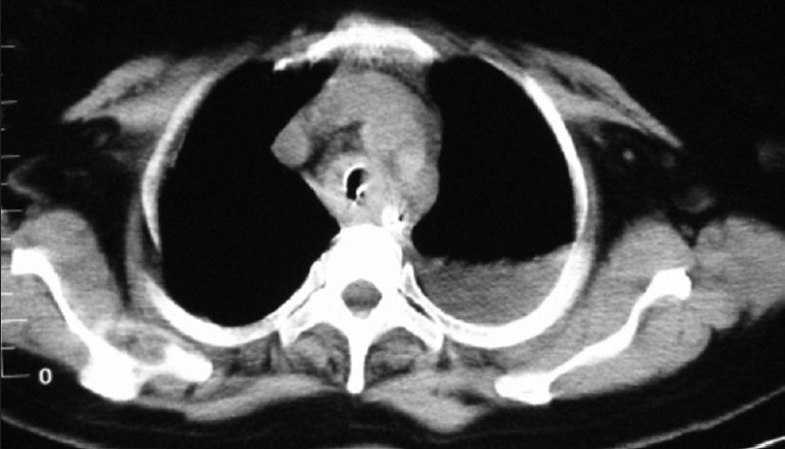
CECT thorax at the level of midtrachea showing diffuse circumferential thickening of tracheal wall with endoluminal growth and left-sided pleural effusion

**Figure 2:**
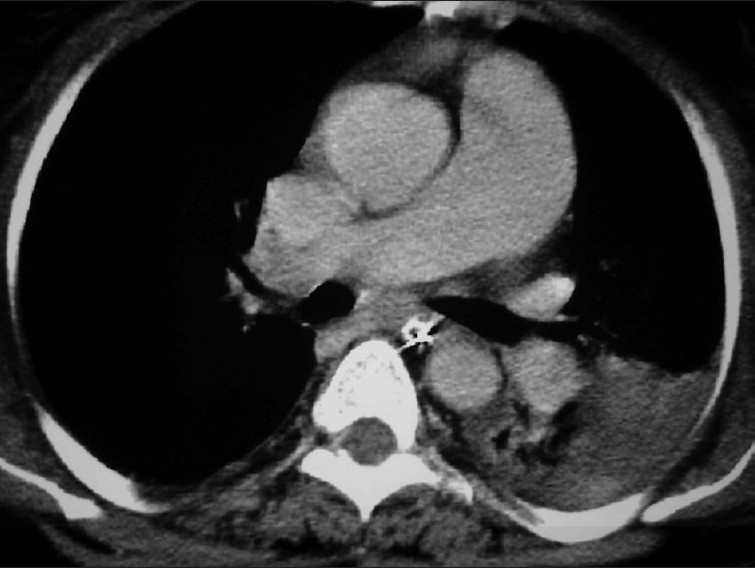
Contrast enhanced CT scan thorax at the level of carina showing consolidation and effusion on left upper lobe

A clinical diagnosis of acute central airway obstruction with obstructive pneumonia and septic shock was made. Contrast-enhanced computed tomography showed heterogeneously enhancing circumferential and diffuse thickening of the tracheal wall with concomitant endoluminal growth in midtracheal region [Figure 1]. An area of consolidation with effusion was also detected in the left upper lobe [Figure 2]. Right tracheobronchial lymph node was enlarged (1.9 × 1.3 cm). No abnormalities were detected in heart and great vessels, upper abdomen, and the adrenal glands.

**Figure 3:**
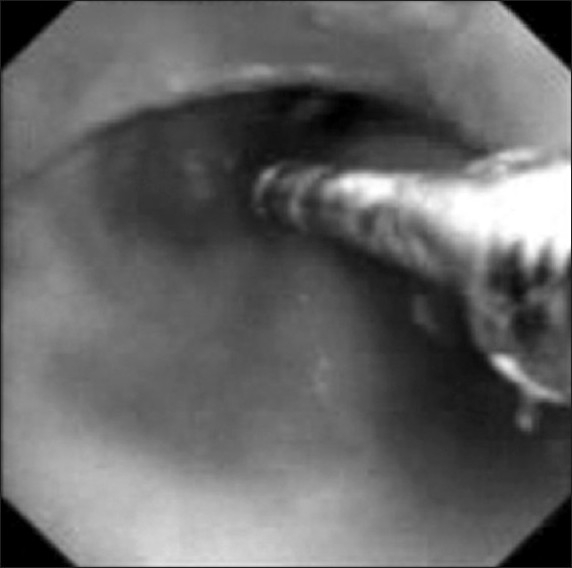
Bronchoscopic image showing submucosal growth in midtrachea with electrocautery probe *in situ*

She underwent repeat bronchoscopy under general anesthesia. Submucosal growth was detected in midtracheal region, occluding more than 80% of tracheal diameter [Figure 3]. The growth was successfully removed by electro-surgery. After the electro-surgery, ventilatory support and inotropic supports were reduced. She was stable for two days, and then she developed progressive drowsiness with deterioration of her clinical condition. She succumbed to death on the third day of hospitalization.

**Figure 4:**
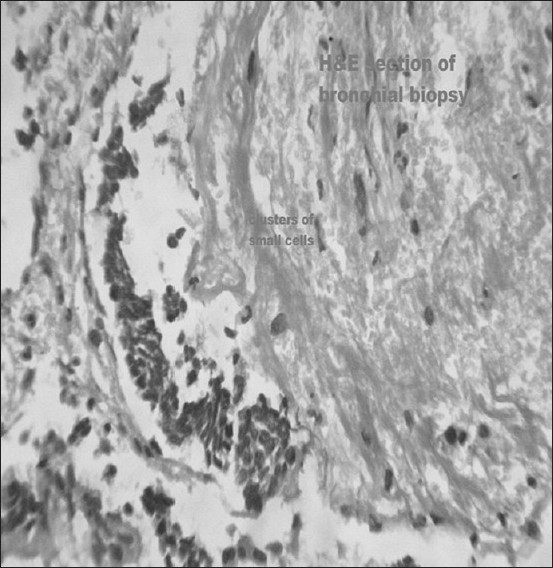
Hematoxylin and eosin stain showing a cluster overcrowded with small hyperchromatic cells with high nuclear-to-cytoplasmic ratio

Histopathological examination of biopsy material showed a cluster overcrowded with small hyperchromatic cells with high nuclear-to- cytoplasmic ratio, suggestive of small cell lung cancer [Figure 4]. Immunohistochemical staining of biopsy material was strongly positive for Neuron specific enolase (NSE) and cytokeratin (CK)-7.

## DISCUSSION

Endotracheal metastasis is relatively uncommon. Most of such metastases are from extrapulmonary malignancy. Tracheal metastases have been reported[1] from tumors of breast, thyroid, nasopharynx, and prostate; colorectal, renal, ovarian, uterine, and testicular tumors; and adrenal carcinomas, sarcomas, melanomas, and plasmacytomas. The overall incidence of metastases from nonpulmonary malignancy is approximately 2%, and the metastases mostly originate from tumors of breast and kidney and colorectal tumors.[2] In the presence of multiple metastases, the incidence is 28%.[3] The mean interval for the appearance of endoluminal lesions after the diagnosis of the primary tumor is usually longer (approximately five years).

Primary lung cancers very rarely metastasize in trachea.[4,5] Chong *et al.,*[5] have reported that the overall prevalence of tracheal metastasis was 0.44% in surgically resected non-small cell lung cancers (0.77% in squamous cell carcinomas and 0.18% in adenocarcinomas) and none in small cell lung cancer in their series.

The exact mechanism of endobronchial metastasis is unknown. The possible mechanism may be due to involvement of peribronchial lymphatics with subsequent progression into the submucosal space.[6] On the basis of developmental mechanisms, Kiryu *et al.,*[1] have classified endobronchial and endotracheal metastases into four categories: Type I, direct metastasis to the bronchus; type II, bronchial invasion by a parenchymal metastatic lesion; type III, bronchial invasion by mediastinal or hilar lymph node metastasis; and type IV, peripheral lesion extending along the proximal bronchus.

The metastatic lesions may be asymptomatic or may present with cough, hemoptysis, dyspnea, and stridor. Bronchoscopy has 100% diagnostic accuracy for identifying endoluminal lesions.

Management of endotracheal metastasis depends upon primary tumor, anatomical location, and the presence of other metastases. Treatment options are surgical excision, endobronchial chemotherapy or radiotherapy, transbronchial laser, photodynamic therapy, or electro-surgery.

The present case demonstrates that small cell lung cancer presented with acute central airway obstruction due to endotracheal metastasis, and the basis of development corresponded to type IV Kiryu classification.
